# Effects of preexisting and new-onset diabetes mellitus on clinical outcomes of patients with heart failure

**DOI:** 10.1080/07853890.2025.2514088

**Published:** 2025-06-05

**Authors:** Ching-Pei Chen, Szu-Chi Chien, Chew-Teng Kor, Che-Ming Hsu

**Affiliations:** a Post-Baccalaureate Medicine, College of Medicine, National Chung Hsing University, Taichung, Taiwan; b Division of Cardiology, Department of Internal Medicine, Changhua Christian Hospital, Changhua, Taiwan; c Graduate Institute of Clinical Medicine, College of Medicine, National Chung Hsing University, Taichung, Taiwan; d Big Data and Digital Promotion Center, Changhua Christian Hospital, Changhua, Taiwan; e Graduate Institute of Statistics and Information Science, National Changhua University of Education, Changhua, Taiwan

**Keywords:** Heart failure, hospitalization for heart failure, major adverse cardiac events, mortality, diabetes Mellitus, inverse propensity score weighting

## Abstract

**Background:**

Diabetes mellitus (DM) is a common comorbidity in heart failure (HF), but the impact of new-onset DM on HF outcomes remains unclear. This study evaluated the effects of DM status on hospitalization for HF (HHF), major adverse cardiac events (MACEs), and mortality in HF patients.

**Methods:**

We conducted a retrospective cohort study of patients newly diagnosed HF at Changhua Christian Hospital, Taiwan, from 2011 to 2021. Patients were grouped as non-DM (*n* = 1477), preexisting DM (*n* = 1488), and new-onset DM (*n* = 328). Inverse propensity score weighting was applied to balance covariates.

**Results:**

Compared to the non-DM group, the preexisting DM was associated with higher risks of HHF [hazard ratio (HR), 1.13; 95% confidence interval (CI), 1.02–1.25], MACEs (HR, 1.22; 95% CI, 1.00–1.49), all-cause mortality (HR, 1.17; 95% CI, 1.01–1.36), and cardiovascular death (HR, 1.54; 95% CI, 1.15–2.06). The new-onset DM group showed a significantly higher risk of HHF (HR, 1.24; 95% CI, 1.01–1.51) and MACEs (HR, 1.22; 95% CI, 1.00–1.49), with nonsignificant trends toward increased all-cause mortality (HR, 1.08; 95% CI, 0.79–1.48) and cardiovascular death (HR, 1.36; 95% CI, 0.74–2.48).

**Conclusion:**

In HF patients, preexisting DM is associated with worse outcomes across multiple endpoints. New-onset DM also elevates risks of HHF and MACE, though its effect on mortality is less clear. Although our study, utilizing electronic medical record data, revealed a different pattern compared to the Danish registry, the findings emphasize the need for individualized management strategies based on DM status in HF care.

## Introduction

Over the past few decades, numerous studies have been conducted on type 2 diabetes mellitus (DM) and heart failure (HF). Several studies have demonstrated that type 2 DM is increasingly recognized as the cause of HF. Older age, coronary artery disease (CAD), peripheral arterial disease, nephropathy, retinopathy, a long duration of DM, obesity, hypertension, and elevated N-terminal prohormone of brain natriuretic peptide (NT-pro-BNP) are all factors that contribute to an increased risk of HF in individuals with diabetes [1–3]. The Framingham Heart Study revealed that DM raises the risk of HF by almost two times in men and by four times in women, even after adjustment for other cardiovascular disease (CVD) risk factors [4].

Besides increasing the risk of HF, DM may also have an adverse effect on the prognosis of HF [5,6], including increased risk of rehospitalization [6], cardiovascular (CV) prognosis [7], CV mortality [8], and one-year mortality [9]. Previous studies have established the adverse effects on the prognosis of patients with HF and diabetes. The relationship between HF and DM was bidirectional and share common pathogenic factors [10,11]. However, limited research exists on the impact of new-onset DM on hospitalization for heart failure (HHF), major adverse cardiovascular events (MACE), and mortality in HF patients. Previous studies have shown that both pre-existing and new-onset diabetes are associated with increased risk, whereas new-onset diabetes has a more pronounced impact on adverse outcomes in HF patients [12]. However, these studies primarily used data from a Danish nationwide cohort, which lacked clinical details such as ejection fraction, heart rate, blood pressure, body mass index, NT-proBNP, and hemoglobin A1c – all of which are important for understanding HF and diabetes. Therefore, this study aimed to compare the risks of clinical outcomes among HF patients with non-DM, preexisting DM, and new-onset DM using electronic medical record (EMR) data.

## Material and methods

### Data source

The research data in this study consisted of patient information retrieved from the Clinical Research Database (CCHRD) of the Changhua Christian Hospital, a tertiary medical center in central Taiwan, which contains data for integrates all EMR pertaining to the heart-failure database, prescriptions, laboratory results, clinical visit records, death records and medical procedures. This study analyzed the data of 3293 heart-failure patients with NT-proBNP ≥ 125 pg/mL who having complete echocardiographic report between 2011- December 2021. This study was conducted in accordance with the Declaration of Helsinki and was reviewed and approved by the Institutional Review Board of Changhua Christian Hospital (IRB No: 220230). As the analyzed data were deidentified and encrypted, the requirement for informed consent was waived under the same IRB protocol (IRB No: 220230).

### Study design and patients

This study analyzed a total of 3293 patients diagnosed with heart failure (ICD-9-CM code 428 or ICD-10-CM code I50) by a cardiologist. All patients had NT-proBNP levels ≥ 125 pg/mL and underwent a complete echocardiographic evaluation. The index date was defined as the date of the first heart failure diagnosis. Patients were categorized into three groups: new-onset DM, preexisting DM, or non-DM, based on the timing of DM diagnosis relative to the index date of HF. Preexisting DM was defined as DM diagnosed prior to the index date of HF, while new-onset DM referred to cases where DM was diagnosed after the index date of HF. Patients without a diagnosis of DM were classified as non-DM. The diagnosis of DM was confirmed using a combination of diabetes-related codes (ICD-9-CM code 250 or ICD-10-CM codes E08–E13) and the prescription of antidiabetic medications.

### Outcome measures and relevant variables

The primary outcomes of interest included hospitalization for heart failure (HHF), major adverse cardiovascular events (MACEs), all-cause mortality, and cardiovascular-related mortality. HHF was defined as a diagnosis of ICD-9-CM code 428 or ICD-10-CM I50 in an inpatient cardiology unit and treated by an attending cardiologist during follow-up. For each case, we thoroughly reviewed the patients’ hospital records, including admission notes, progress notes, and discharge summaries, to confirm that heart failure was the primary reason for hospitalization. MACEs were a composite of acute myocardial infarction (ICD-9-CM code 410 or ICD-10-CM I21) and ischemic stroke (ICD-9-CM code 433, 434, 436 or ICD-10-CM I60–I63). Death events were identified using death records from the CCHRD. Cardiovascular-related death was defined as any death attributed to a cardiovascular condition, based on a thorough review of documented causes and relevant clinical information. Cardiovascular death was defined based on specific causes directly linked to cardiovascular pathology, including but not limited to—cardiogenic shock, acute myocardial infarction, heart failure (including acute decompensated heart failure), arrhythmias (e.g. ventricular fibrillation, ventricular tachycardia, or sudden cardiac arrest), pulmonary edema, and other cardiovascular complications such as valvular heart disease or myocarditis.

The demographic and clinical information of patients, including age, gender, BMI, LVEF, heart failure phenotype based on ejection fraction, pre-index date medications, serum biochemical data and presence of comorbidities, was collected from the CCHRD. Comorbidities included hypertension, hyperlipidemia, coronary artery disease, chronic obstructive pulmonary disease (COPD), chronic kidney disease (CKD), atrial fibrillation, and stroke. Medications included antihypertensive agents (ACE inhibitors/ARBs, alpha blockers, beta blockers, calcium channel blockers, thiazides, loop diuretics and spironolactone), and statins. All patients were followed from the index date until the date of HHF, MACEs, or death, or the end of 2021, whichever occurred first. Death was considered a competing event for HHF and MACEs.

## Statistical analysis

Continuous variables were summarized as medians with interquartile ranges (IQR), while categorical variables were presented as counts and percentages. Statistical comparisons were performed using the Kruskal-Wallis H test for continuous variables and either the chi-squared test or Fisher’s exact test for categorical variables, depending on the data distribution.

To address potential confounding due to imbalanced baseline characteristics across the three study groups, inverse probability weighting (IPW) was applied. Propensity scores were calculated using multinomial logistic regression [13], incorporating all baseline characteristics to estimate the probability of belonging to either the preexisting DM group or the new-onset DM group. These probabilities were then converted into weights by taking their inverse values to achieve covariate balance across the groups.

Given that DM is a time-varying exposure, the Simon and Makuch method [14] was used as an alternative to Kaplan-Meier estimation to visualize the cumulative incidence rates of study outcomes across the three groups. To address immortal time bias in the new-onset DM group and account for the competing risk of death, IPW-adjusted time-dependent cause-specific Cox proportional hazards models were applied to assess the association between DM status and the risk of study outcomes. The statistical techniques employed in this study were consistent with those used in previous research [15–17]. Results were presented as hazard ratios (HRs) with 95% confidence intervals (CIs). To assess the robustness of the findings, sensitivity analyses were performed by excluding patients with preexisting cardiovascular conditions, such as coronary artery disease (CAD), stroke, atrial fibrillation (AF), or dysrhythmia. Additionally, to reduce misclassification bias and enhance diagnostic accuracy, a second sensitivity analysis was performed using glycated hemoglobin (HbA1c) ≥6.5% as an additional criterion for defining diabetes. Subgroup analyses were also performed to examine the relationship between DM status and clinical outcomes, stratified by age (using a 75-year-old cutoff) and heart failure (HF) phenotype based on ejection fraction. Additionally, factors associated with new-onset DM in non-DM patients with HF were identified.

All statistical analyses were conducted using SAS software (version 9.4), and visualization plots were created using R software (version 4.1.0; The Comprehensive R Archive Network: http://cran.r-project.org). A two-tailed p-value of <0.05 was considered statistically significant.

## Results

### Patient characteristics

**Figure 1.**
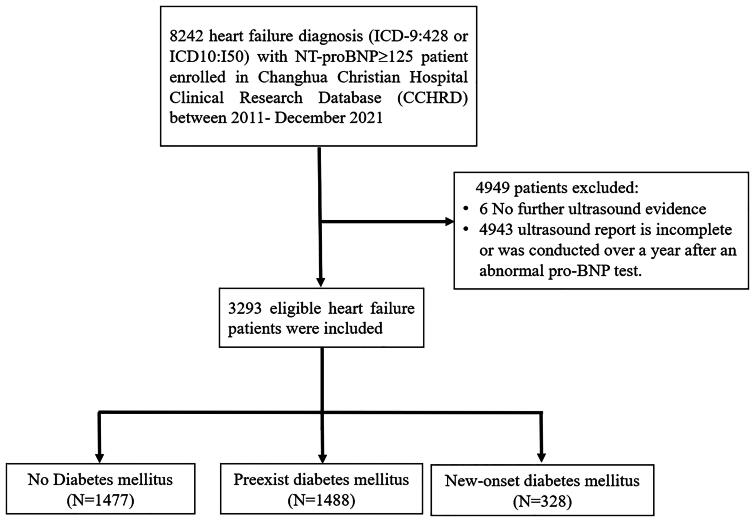
Flowchart of patient selection.

The flowchart of the patient selection process is shown in Figure 1. After ineligible patients were excluded, 3293 patients with incident HF were enrolled and were classified as non-DM group (*n* = 1477), preexisting DM group (*n* = 1488), and new-onset DM group (*n* = 328). Table 1 shows the baseline characteristics of the study patients stratified using DM status. Before the IPW process, statistically significant differences were found between DM groups across almost baseline covariates (see Table 1). After IPW, all covariates were well balanced between the DM groups.

**Table 1.** Demographic and clinical characteristics of patients with heart failure.

	No DM	Pre-existing DM	New onset DM	Total	*P*-value before IPTW	*P*-value after IPTW
Sample size	1477	1488	328	3293		
LVEF	44 (29, 62)	44 (31, 62)	39 (27, 58)	43 (30, 62)	0.001	0.503
Heart failure phenotype						
HFrEF	657 (44.5%)	646 (43.4%)	179 (54.6%)	1482 (45%)	0.002	0.472
HFmrEF	212 (14.4%)	198 (13.3%)	45 (13.7%)	455 (13.8%)		
HFpEF	608 (41.2%)	644 (43.3%)	104 (31.7%)	1356 (41.2%)		
Age	77 (62, 83)	74 (64, 82)	72 (59, 81)	75 (63, 82)	<0.001	0.926
Gender, male	806 (54.6%)	764 (51.3%)	191 (58.2%)	1761 (53.5%)	0.041	0.741
BMI	24.1 (21.2, 27.1)	25.5 (22.9, 28.5)	25.5 (23.0, 28.4)	25.0 (22.0, 27.9)	<0.001	0.068
NT pro-BNP	4135 (1668, 9145)	4532.5 (1788, 10362.5)	3691.5 (1593, 9045.5)	4312 (1700, 9663)	0.009	0.496
Comorbidity disease						
Hypertension	628 (42.5%)	1038 (69.8%)	92 (28%)	1758 (53.4%)	<0.001	0.917
Hyperlipidemia	247 (16.7%)	691 (46.4%)	42 (12.8%)	980 (29.8%)	<0.001	0.498
Coronary artery disease	334 (22.6%)	548 (36.8%)	35 (10.7%)	917 (27.8%)	<0.001	0.161
COPD	214 (14.5%)	247 (16.6%)	30 (9.1%)	491 (14.9%)	0.002	0.884
CKD	214 (14.5%)	490 (32.9%)	21 (6.4%)	725 (22%)	<0.001	0.699
Atrial fibrillation	382 (25.9%)	382 (25.7%)	41 (12.5%)	805 (24.4%)	<0.001	0.416
Stroke	200 (13.5%)	356 (23.9%)	26 (7.9%)	582 (17.7%)	<0.001	0.738
Medication use in hypertension						
ACEARB	1174 (79.5%)	1265 (85%)	296 (90.2%)	2735 (83.1%)	<0.001	0.884
Alpha blocker	75 (5.1%)	149 (10%)	29 (8.8%)	253 (7.7%)	<0.001	0.781
Beta blocker	1007 (68.2%)	1112 (74.7%)	232 (70.7%)	2351 (71.4%)	<0.001	0.592
Calcium channel blocker	712 (48.2%)	894 (60.1%)	192 (58.5%)	1798 (54.6%)	<0.001	0.252
Thiazide	140 (9.5%)	202 (13.6%)	50 (15.2%)	392 (11.9%)	<0.001	0.415
Loop diuretics	1258 (85.2%)	1318 (88.6%)	288 (87.8%)	2864 (87%)	0.020	0.969
Spironolactone	791 (53.6%)	735 (49.4%)	186 (56.7%)	1712 (52%)	0.015	0.894
Statin	392 (26.5%)	775 (52.1%)	139 (42.4%)	1306 (39.7%)	<0.001	0.514
Lab data						
HbA1c	5.8 (5.4, 6.1)	6.8 (6.1, 7.7)	6.1 (5.7, 6.2)	6.3 (5.7, 7.1)	<0.001	0.236
Hb	12.3 (10.4, 13.8)	11 (9.3, 12.9)	12.5 (10.5, 14.3)	11.8 (9.8, 13.4)	<0.001	0.394
WBC count	7.9 (6.1, 10.4)	8.7 (6.8, 11.8)	8.9 (6.6, 11.4)	8.4 (6.5, 11.1)	<0.001	0.420
Estimated GFR	56.9 (39.9, 76.5)	42.7 (19.6, 64.3)	52.4 (33.8, 68.8)	50.9 (29.4, 70.8)	<0.001	0.535
Triglyceride	106 (73.6, 140)	117 (83, 162.3)	124 (86.6, 160.1)	113 (80, 152)	<0.001	0.124
LDL cholesterol	88.5 (74.6, 104.4)	86 (70.3, 104)	96.6 (79.1, 110.8)	88 (73.4, 105)	<0.001	0.440
Outcome						
HHF	668 (45.2%)	807 (54.2%)	220 (67.1%)	1695 (51.5%)	<0.001	<0.001
MACE	161 (10.9%)	222 (14.9%)	70 (21.3%)	453 (13.8%)	<0.001	0.005
All-cause mortality	422 (28.6%)	478 (32.1%)	79 (24.1%)	979 (29.7%)	0.007	0.135
CV-related mortality	98 (6.6%)	132 (8.9%)	24 (7.3%)	254 (7.7%)	0.071	0.115

Abbreviations: CV: cardiovascular; COPD: chronic obstructive pulmonary disease; CKD: chronic kidney disease.

^a^ Inverse probability of group-weighted (IPW) was estimated by the propensity score from generalized boosted regression.

* MACEs = the composite of acute myocardial infarction and ischemic stroke.

Generally, patients with preexisting DM had the highest proportion of mortality and CV-related death, followed by the new-onset DM group and then the non-DM group. Notably, the new-onset DM group had the highest proportion of HHF and MACE.

### Long-term HHF risk based on diabetes mellitus status

**Figure 2.**
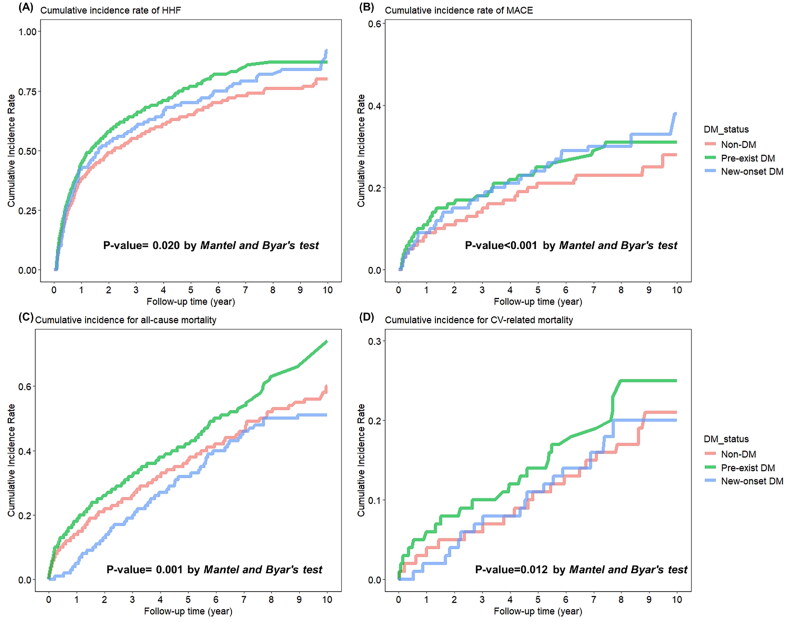
Kaplan–Meier curves for cumulative incidences of clinical outcomes between preexisting DM, new-onset DM and non-DM cohorts of patients with heart failure (HF) for (A) hospitalization for HF (HHF), (B) major adverse cardiac events (MACEs), (C) all-cause mortality and (D) cardiovascular (CV)-related mortality.

The incidence rates of HHF for the preexisting DM, new-onset DM, and non-DM groups were 39.22, 52.60 and 24.12 per 1000 person-years, respectively, as shown in Table 2. The cumulative HHF probability for patients in the three DM groups using the Simon and Makuch method is shown in Figure 2A. In multivariate unweighted time-dependent Cox models, both preexisting and new-onset DM were associated with a higher risk of HHF compared to the no DM group (preexisting DM: aHR 1.14, 95% CI 1.02–1.26, *p* = 0.018; new-onset DM: aHR 1.21, 95% CI 1.01–1.46, *p* = 0.042). Similarly, in multivariate IPW-adjusted cause-specific and time-dependent Cox models, preexisting DM was significantly associated with a 1.13-fold increased risk of HHF (aHR 1.13, 95% CI 1.02–1.25, *p* = 0.017), while new-onset DM was associated with a 1.24-fold increased risk of HHF (aHR 1.24, 95% CI 1.01–1.51, *p* = 0.036) compared to the non-DM group.

**Table 2.** Risks for (A) HHF, (B) MACE, (C) all-cause mortality and (D) CV-related mortality among patients with HF by DM status.

	Unweighted incidence rate		Unweight time-dependent Cox Model^†^				Weight time-dependent Cox Model^†^	
	Event	IR (95% CI)	cHR (95% CI)	*p*-value	aHR (95% CI)	*p*-value	aHR (95% CI)	*p*-value
*Hospitalization for heart-failure*								
Non-DM	668	24.12 (22.29, 25.95)	1		1		1	
Pre-existing DM	807	39.22 (36.51, 41.92)	1.33 (1.21, 1.47)	<0.001	1.14 (1.02, 1.26)	0.018	1.13 (1.02, 1.25)	0.017
New onset DM	220	52.60 (45.65, 59.55)	1.32 (1.10, 1.58)	0.003	1.21 (1.01, 1.45)	0.042	1.24 (1.01, 1.51)	0.036
*MACE*‡								
Non-DM	161	4.48 (3.79, 5.17)	1		1		1	
Pre-existing DM	222	7.02 (6.10, 7.95)	1.42 (1.17, 1.73)	0.001	1.24 (1.00, 1.54)	0.050	1.22 (1.00, 1.49)	0.054
New onset DM	70	8.85 (6.78, 10.92)	1.82 (1.34, 2.48)	<0.001	1.70 (1.24, 2.33)	0.001	1.65 (1.14, 2.40)	0.008
*All-cause mortality*								
Non-DM	422	10.97 (9.93, 12.02)	1		1		1	
Pre-existing DM	478	13.51 (12.30, 14.72)	1.25 (1.10, 1.43)	0.001	1.26 (1.09, 1.45)	0.002	1.17 (1.01, 1.36)	0.032
New onset DM	79	8.12 (6.33, 9.91)	0.92 (0.72, 1.18)	0.528	1.05 (0.82, 1.35)	0.680	1.08 (0.79, 1.48)	0.617
*Cardiovascular death*								
Non-DM	98	2.55 (2.04, 3.05)	1		1		1	
Pre-existing DM	132	3.73 (3.09, 4.37)	1.48 (1.14, 1.93)	0.003	1.47 (1.12, 1.93)	0.006	1.54 (1.15, 2.06)	0.003
New onset DM	24	2.47 (1.48, 3.45)	1.19 (0.76, 1.89)	0.448	1.28 (0.81, 2.03)	0.297	1.36 (0.74, 2.48)	0.322

Abbreviations: CI: confidence interval; aHR: adjusted hazard ratio; IR: incidence rate (per 1000 person-years); HF: heart-failure; MACEs: major adverse cardiac events.

^†^ aHR was calculated from IPW-standardized time dependent cause-specific Cox model, where the inverse probability of group-weighted (IPW) was estimated by the propensity of group from multinomial logistic regression.

^‡^ MACE = the composite of acute myocardial infarction and ischemic stroke.

### Long-term MACE risk based on diabetes mellitus status

The incidence rates of MACE for the preexisting DM, new-onset DM, and non-DM groups were 7.02, 8.85 and 4.48 per 1000 person-years, respectively, as shown in Table 2. The cumulative MACE probability for patients in the three DM groups using the Simon and Makuch method is shown in Figure 2B. In multivariate unweighted time-dependent Cox models, both preexisting and new-onset DM were associated with a higher risk of MACE compared to the no DM group (preexisting DM: aHR 1.24, 95% CI 1.00–1.54, *p* = 0.050; new-onset DM: aHR 1.70, 95% CI 1.24–2.33, *p* = 0.001). In multivariable IPW-adjusted cause-specific and time-dependent Cox models, preexisting DM was borderline associated with a 1.22-fold higher risk of MACE (aHR, 1.22, 95% CI, 1.00–1.49, *p*-value = 0.054), whereas new-onset DM was associated with a 1.65-fold higher risk of MACE (aHR, 1.65; 95% CI, 1.14–2.40, *p*-value = 0.008) when compared with the non-DM group.

### Long-term mortality risk based on diabetes mellitus status

The incidence rates of all-cause mortality and CV-death in the preexisting DM, new-onset DM, and non-DM groups were 13.51 and 3.73, 8.12 and 2.47 and 10.97 and 2.55 per 1000 person-years, respectively (Table 2). The cumulative incidence of all-cause mortality and CV-death was plotted for the DM groups in Figure 2C and D. In multivariate unweighted time-dependent Cox models, preexisting DM were associated with a higher risk of all-cause death and CV-death compared to the no DM group (all-cause mortality: aHR 1.26, 95% CI 1.09–1.45, *p* = 0.002; CV-death: aHR 1.47, 95% CI 1.12–1.93, *p* = 0.006). However, new-onset DM were not associated with the risk of all-cause death and CV-death compared to the non-DM group (all-cause mortality: aHR 1.08, 95% CI 0.79–1.48, *p* = 0.617; CV-death: aHR 1.36, 95% CI 0.74–2.48, *p* = 0.322). In multivariable IPW-adjusted cause-specific and time-dependent Cox models (Table 2), preexisting DM was significantly associated with 1.17-fold and 1.54-fold higher risks of all-cause mortality and CV-death compared with non-DM (all-cause mortality: aHR 1.17, 95% CI 1.01–1.36, *p* = 0.032; CV-death: aHR 1.54, 95% CI 1.15–2.06, *p* = 0.003), respectively. New-onset DM was not significantly associated with 1.08-fold and 1.36-fold higher risks of all-cause mortality and CV-death compared with non-DM (all-cause mortality: aHR 1.08, 95% CI 0.79–1.48, *p* = 0.617; CV-death: aHR 1.36, 95% CI 0.74–2.48, *p* = 0.322), respectively.

## Sensitivity analysis

The sensitivity analysis results are presented in Table 3. In the first sensitivity test, patients with a history of coronary artery disease (CAD), stroke, atrial fibrillation, or dysrhythmia were excluded. The analysis showed that both preexisting and new-onset DM groups were associated with a higher risk of HHF. Preexisting DM was borderline associated with a 1.21-fold increased risk of MACE (aHR, 1.21; 95% CI, 0.99–1.48; *p* = 0.065), while new-onset DM was significantly associated with a 1.60-fold increased risk of MACE (aHR, 1.60; 95% CI, 1.11–2.33; *p* = 0.013) compared to the non-DM group. Preexisting DM was also associated with a higher risk of all-cause death and CV-death compared to the non-DM group. However, new-onset DM was not associated with an increased risk of all-cause death or CV-death compared to the non-DM group. The associations between different DM statuses and clinical outcomes remained consistent with the main analyses after excluding patients with a history of CAD, stroke, atrial fibrillation, or dysrhythmia, demonstrating the robustness of our findings.

**Table 3.** Sensitivity analysis for clinical outcome.

	Hospitalization for heart-failure		MACE		All-cause mortality		Cardiovascular death	
	aHR (95% CI)	*p*-value	aHR (95% CI)	*p*-value	aHR (95% CI)	*p*-value	aHR (95% CI)	*p*-value
Excluding patients with preexisting cardiovascular conditions								
Non-DM	1		1		1		1	
Pre-existing DM	1.13 (1.02, 1.25)	0.017	1.21 (0.99, 1.48)	0.065	1.17 (1.01, 1.36)	0.032	1.54 (1.15, 2.06)	0.003
New onset DM	1.24 (1.01, 1.51)	0.036	1.60 (1.11, 2.33)	0.013	1.08 (0.79, 1.48)	0.617	1.36 (0.74, 2.48)	0.322
Using glycated hemoglobin (HbA1c) ≥6.5% as an additional criterion for defining diabetes								
Non-DM	1		1		1		1	
Pre-existing DM	1.26 (1.12, 1.42)	<0.001	1.32 (1.05, 1.67)	0.019	1.22 (1.04, 1.44)	0.016	1.21 (0.92, 1.58)	0.169
New onset DM	1.44 (1.14, 1.83)	0.003	1.64 (1.07, 2.51)	0.024	0.86 (0.6, 1.23)	0.413	1.20 (0.72, 1.99)	0.485

To improve diagnostic accuracy, glycated hemo­globin (HbA1c) ≥6.5% as an additional criterion for defining diabetes. Among patients classified as having diabetes, 88.51% of those with pre-existing diabetes and 84.76% of those with new-onset diabetes met this HbA1c threshold. The results of second sensitivity analysis were also consistent with the primary analysis, with one notable distinction: pre-existing diabetes was associated with a significantly increased risk of MACE (aHR, 1.32; 95% CI, 1.05–1.67; *p* = 0.019) but was not significantly associated with cardiovascular death (aHR, 1.21; 95% CI, 0.92–1.58; *p* = 0.169).

### Subgroup analysis

Subgroup analysis was conducted to investigate potential interactions between DM status, age, and EF type on clinical outcomes in Table 4. The analysis showed no significant differences in the association between DM status and clinical outcomes when stratified by age and EF type, as indicated by the non-significant p-value for the interaction term between DM status and the subgroups stratified by age and EF type. These findings suggest that DM status may serve as a potential risk factor for clinical outcomes, independent of age and EF type.

**Table 4.** Subgroup analysis for assessing the interaction effect of DM status, age and EF type on clinical outcome.

Subgroup	HHF		MACE		Death		CV mortality	
	Pre-existing DM	New onset DM	Pre-existing DM	New onset DM	Pre-existing DM	New onset DM	Pre-existing DM	New onset DM
	aHR (95% CI)	aHR (95% CI)	aHR (95% CI)	aHR (95% CI)	aHR (95% CI)	aHR (95% CI)	aHR (95% CI)	aHR (95% CI)
**Age**								
<75	1.15 (0.99, 1.34)	1.60 (1.18, 2.18)	1.26 (0.93, 1.70)	2.01 (1.23, 3.30)	1.09 (0.86, 1.38)	1.53 (0.97, 2.41)	1.87 (1.18, 2.98)	1.49 (0.65, 3.45)
≥75	1.22 (1.06, 1.39)	0.96 (0.73, 1.25)	1.09 (0.83, 1.44)	1.01 (0.61, 1.68)	1.35 (1.15, 1.58)	0.98 (0.72, 1.33)	1.68 (1.21, 2.35)	2.14 (1.27, 3.59)
P interaction	0.735	0.259	0.5517	0.1681	0.2361	0.8819	0.6888	0.2490
**EF**								
HFrEF (*n* = 1482)	1.07 (0.93, 1.24)	1.18 (0.87, 1.60)	1.45 (1.07, 1.97)	1.88 (1.06, 3.31)	1.34 (1.08, 1.65)	1.17 (0.77, 1.79)	1.81 (1.26, 2.6)	2.24 (1.20, 4.20)
HFmrEF (*n* = 455)	1.16 (0.89, 1.51)	1.77 (1.06, 2.97)	1.31 (0.82, 2.09)	1.03 (0.42, 2.56)	1.01 (0.68, 1.49)	1.35 (0.69, 2.62)	1.88 (0.87, 4.09)	5.5 (2.17, 13.95)
HFpEF (*n* = 1356)	1.18 (1.00, 1.38)	1.27 (0.93, 1.74)	0.86 (0.62, 1.18)	1.21 (0.67, 2.16)	1.16 (0.95, 1.40)	0.98 (0.70, 1.37)	1.28 (0.79, 2.07)	0.75 (0.29, 1.92)
P interaction	0.4095	0.7958	0.0703	0.2762	0.5785	0.7830	0.1540	0.1594

Abbreviations: CI: confidence interval; aHR: adjusted hazard ratio; HHF: Hospitalization for heart-failure; MACEs: major adverse cardiac events; CV: cardiovascular; DM: Diabetes mellitus.

### Significant factors for new-onset diabetes mellitus in non-diabetic heart failure patients

The risk factors for the development of new-onset DM in non-Diabetic patients (*n* = 1805) were shown in Table 5. Factors that positively contributed to new-onset DM included the higher HbA1c and triglyceride, presence of HFrEF, and the use of ACEi/ARB, calcium channel blocker, thiazide, statin. Higher eGFR is negatively contributed to new-onset DM.

**Table 5.** The significant variables associated with the development of new-onset DM in HF patients without DM at baseline (*N* = 1805).

Variables	Adjusted HR (95% CI)	*p*-value
**EF**		
HFrEF	1.41 (1.09, 1.83)	0.008
HFmrEF	1.22 (0.85, 1.74)	0.275
HFpEF	1	
ACEi/ARB	1.76 (1.19, 2.61)	0.004
Calcium channel blocker	1.45 (1.15, 1.82)	0.002
Thiazide	1.48 (1.09, 2.01)	0.012
Statin	1.81 (1.44, 2.27)	<0.001
HbA1c	1.47 (1.35, 1.60)	<0.001
eGFR	0.93 (0.90, 0.97)	0.001
Triglyceride	1.03 (1.01, 1.04)	0.002

### Bidirectional relationship between DM and HF hemodynamic phenotypes

We used the algorithm developed by Stevenson et al. [18] to classify HF patients based on their clinical-hemodynamic phenotype. In our study, the distribution of clinical-hemodynamic phenotype among HF patients was as follows: 20.5% for dry-warm phenotype, 64.53% for wet-warm phenotype, 12.33% for wet-cold phenotype, and 2.64% for dry-cold phenotype. The wet-warm phenotype was the most common phenotype, while the dry-cold phenotype was the least frequent, consistent with findings from previous studies [19]. We conducted two separate analyses to further explore the bidirectional relationship between DM and HF hemodynamic phenotypes. First model evaluated the impact of preexisting DM on the hemodynamic phenotypes of HF (Table S1). Compared to patients without preexisting DM, those with DM showed no significant association with the wet-warm phenotype (adjusted odds ratio [aOR], 1.16; 95% CI, 0.95–1.42; *p* = 0.140) or the dry-cold phenotype (aOR, 1.33; 95% CI, 0.81–2.19; *p* = 0.264). However, preexisting DM was significantly associated with the wet-cold phenotype (aOR, 1.46; 95% CI, 1.10–1.93; *p* = 0.009), indicating that patients with DM are more likely to present with both congestion and hypoperfusion. These findings suggest that preexisting DM may contribute to more severe hemodynamic impairment in HF patients.

Second model assessed the impact of HF hemodynamic phenotypes on the development of new-onset DM (Table S2). Using the dry-warm phenotype as the reference group, no significant associations were found between any HF hemodynamic phenotypes and the development of new-onset DM. These findings suggest that HF hemodynamic phenotypes may not be strongly associated with the risk of developing new-onset DM.

## Discussion

To the best of our knowledge, this is the first study to investigate the impact of DM status (non-DM, preexisting DM, and new-onset DM) on clinical outcomes in patients with HF, utilizing electronic medical records that include detailed clinical variables related to HF and diabetes. This study found that, compared to HF patients without DM, the preexisting of DM was associated with higher risks of HHF, MACEs, all-cause mortality, and CV-death. However, there was an increased risk of HHF and MACEs in the new-onset DM group, but no significant association with all-cause mortality or CV-death.

DM is a major disease concomitant with HF worldwide. In the United States, more than 29 million adults have type 2 DM [20], whereas 6.5 million have HF [21]. In Taiwan, 43.6% of patients with HF have type 2 DM [22]. Studies have shown that patients with both HF and DM have poorer clinical outcomes compared to those with HF alone. Observational studies using heart failure registry databases indicate that the presence of DM in patients with HF increases the risk of death in both hospitalized and ambulatory HF patients [5,9]. In addition to mortality, DM adversely affects the prognosis of patients with HF [23]. Patients with DM have a 50% higher risk of hospitalization compared with non-diabetic patients. Additionally, patients with DM had modestly higher rates of hospital readmission [24]. Finally, patients with both DM and HF have poorer health-related quality of life compared with patients with HF alone [25,26].

Previous studies have established the association between preexisting DM and adverse clinical outcomes among HF patients, but evidence regarding the role of new-onset DM remains limited. Zareini et al. [12] investigated the impact of new-onset DM on mortality among 104,522 HF patients using 12 years of Danish registry data. Their findings demonstrated that, compared to patients without DM, new-onset DM was associated with a higher risk of death, while preexisting DM was associated with an intermediate risk. In contrast, our study, which utilized EMR data, revealed a different pattern. Specifically, we found that preexisting DM was associated with worse mortality outcomes in HF patients, whereas new-onset DM was not significantly associated with mortality outcomes. While both our study and Zareini et al. [12] considered changes in DM status over time, a key distinction of our study is its focus on additional HF clinical outcomes, such as HHF and MACEs. Notably, new-onset DM in our study was significantly associated with an increased risk of both HHF and MACEs. These findings provide more robust evidence supporting the association between new-onset DM and adverse clinical outcomes in HF patients. Our results highlight the importance of regular blood glucose monitoring in HF patients without DM to enable early detection of new-onset DM. Furthermore, HF patients with concurrent DM, including its long-term complications and potential impact on HF progression, require comprehensive medical care to address these two comorbidities effectively.

HF and DM share a bidirectional relationship, with new-onset diabetes (often referred to as cardiogenic diabetes) being common in HF patients due to increased insulin resistance and a reduced insulin secretory response of pancreatic β-cells to hyperglycemia [27,28]. Guglin et al. demonstrated that HF more than doubles the incidence of diabetes in the elderly population [10]. They also reported that increasing HF severity, as assessed by loop diuretic requirements, was associated with a higher risk of developing DM. In our study, we classified HF phenotypes using left ventricular ejection fraction (LVEF) based on the European Society of Cardiology criteria [29]. We found that patients with HFrEF had a 1.41-fold increased incidence of DM, and those with HFmrEF had a 1.22-fold increased incidence, compared to patients with HFpEF. Scherbakov et al. reported that patients with HFrEF exhibit more severe insulin resistance (IR) [30], which may contribute to the development of diabetes. Our study builds on these findings by demonstrating, through a large-scale cohort analysis using an EMR dataset, that HFrEF is associated with an increased risk of developing diabetes. While previous studies, such as those by Guglin et al. [10], have highlighted the relationship between HF and the development of DM, no prior research has specifically investigated the impact of different HF phenotypes on DM risk.

In addition to pathophysiologic phenotypes, recent evidence has classified HF into clinical-hemodynamic phenotypes based on the presence of hypoperfusion and congestion [29,31]. In this study, we investigated the bidirectional relationship between DM and HF hemodynamic phenotypes. Our findings revealed distinct associations between DM, the wet-cold hemodynamic phenotype, and HFrEF. Preexisting DM was significantly associated with the wet-cold phenotype, defined by both congestion and hypoperfusion, suggesting that DM may contribute to worsening hemodynamic compromise in HF. Conversely, no significant associations were observed between HF hemodynamic phenotypes and the risk of developing new-onset DM. However, HFrEF was significantly associated with an increased risk of incident DM, highlighting the potential role of underlying HF pathophysiology in promoting metabolic dysfunction. These findings suggest that while DM may influence hemodynamic status in HF, hemodynamic phenotypes alone may not be strong predictors of new-onset DM. To our knowledge, this is the first study to explore the bidirectional relationship between DM, HF pathophysiologic phenotypes, and HF hemodynamic phenotypes.

In line with findings of previous studies, we observed that elevated HbA1c [32], triglycerides (TG) [33], and lower estimated glomerular filtration rate (eGFR) [34] are significant contributors to the development of DM.

In our study, we observed that certain medications, including ACE inhibitors/ARBs, calcium channel blockers (CCBs), thiazides, and statins, were associated with an increased risk of new-onset DM. Previous studies have confirmed that thiazide causes glucose intolerance and related adverse metabolic events, including new-onset DM [35,36]. However, we acknowledge that some of these findings, particularly those related to CCBs and ACE inhibitors/ARBs, may be difficult to interpret due to the potential influence of confounding factors, highlighting the inherent limitations of observational studies in establishing causal relationships.

This study has several limitations. First, the use of an EMR database from a single center may limit the generalizability of the findings to broader populations. Second, factors such as income, dietary habits, smoking habits, and alcohol consumption, which may contribute to glycemic control and HF progression, were not included in our analysis due to the absence of these variables in the dataset. Third, the retrospective cohort design of our study makes it challenging to establish causal relationships between variables. Although we employed IPW with propensity score analysis to adjust for potential confounding factors, further prospective studies are needed to validate our findings and establish causality.

## Conclusions

This large-scale, long-term electronic medical record data demonstrated heart failure patients with preexisting diabetes mellitus had a broad adverse impact, significantly increasing risks of heart failure hospitalization, MACE, all-cause mortality, and cardiovascular-related death. In contrast, new-onset diabetes mellitus may increase the risk of heart failure hospitalization and MACE, without significant effects on mortality outcomes. These findings have important clinical implications for managing HF patients with pre-existing and new-onset DM. Patients with pre-existing DM may require closer cardiovascular monitoring and more intensive risk management. This underscores the need for early and comprehensive cardiovascular risk assessment, including tighter glycemic control, optimization of HF therapies, and regular multidisciplinary follow-ups to mitigate adverse outcomes. Additionally, the strong association between HFrEF and new-onset DM highlights the importance of proactive metabolic screening and early intervention. Clinicians should implement preventive strategies such as lifestyle modifications, weight management, and careful selection of HF therapies that minimize hyperglycemia risk. Overall, these findings reinforce the need for a personalized, proactive approach to HF management in patients with DM. By addressing both cardiovascular and metabolic risks in a coordinated manner, clinicians can improve outcomes and reduce complications in this high-risk population.

## Supplementary Material

Supplementary_tables.docx

## Data Availability

The data that support the findings of this study originate from Changhua Christian Hospital clinical research database. Restrictions apply to the availability of these data and they are therefore not publicly available. The datasets used and/or analyzed during the current study are available from the corresponding author on reasonable request.
