# Supplementary material for: Effects of preexisting and new-onset diabetes mellitus on clinical outcomes of patients with heart failure
Source: Ann Med. 2025 Jun 5;57(1):2514088. doi: 10.1080/07853890.2025.2514088 (PMC12143004; doi:10.1080/07853890.2025.2514088)
Supplement: Supplementary_tables.docx [file IANN_A_2514088_SM7425.docx]

**Supplementary Tables**

Table S1: Evaluated the impact of preexisting DM on the hemodynamic phenotypes of HF.

| Model 1: preexisting DM🡪 phenotypes of HF | | | | | | |
| --- | --- | --- | --- | --- | --- | --- |
| Pre-existing DM | Wet-warm | | Wet-cold | | Dry-cold | |
|  | aOR (95% CI) | P-value | aOR (95% CI) | P-value | aOR (95% CI) | P-value |
| No | 1 |  | 1 |  | 1 |  |
| Yes | 1.16(0.95, 1.42) | 0.140 | **1.46(1.10,1.93)** | **0.009** | 1.33(0.81, 2.19) | 0.264 |

Table S2: Assessed the impact of HF hemodynamic phenotypes on the development of new-onset DM.

| Model 2: Phenotypes of HF🡪 the development of new-onset DM | | |
| --- | --- | --- |
| Phenotype | aHR (95% CI) | P-value |
| Dry-warm | 1 |  |
| **Wet-warm** | 0.80(0.62,1.03) | 0.083 |
| **Wet-cold** | 0.73(0.48,1.11) | 0.143 |
| Dry-cold | 0.53(0.22,1.26) | 0.152 |
